# GPR30 Deficiency Causes Increased Bone Mass, Mineralization, and Growth Plate Proliferative Activity in Male Mice

**DOI:** 10.1002/jbmr.209

**Published:** 2010-08-23

**Authors:** Jeffery Ford, Asghar Hajibeigi, Michael Long, Lisa Hahner, Crystal Gore, Jer-Tseng Hsieh, Deborah Clegg, Joseph Zerwekh, Orhan K Öz

**Affiliations:** 1 Department of Radiology, University of Texas Southwestern Medical Center at Dallas Dallas, TX, USA; 2 Department of Urology, University of Texas Southwestern Medical Center at Dallas Dallas, TX, USA; 3 Department of Internal Medicine, University of Texas Southwestern Medical Center at Dallas Dallas, TX, USA

**Keywords:** GPR30, BONE, OSTEOBLAST, BONE DENSITY, GROWTH PLATE

## Abstract

Estrogen regulation of the male skeleton was first clearly demonstrated in patients with aromatase deficiency or a mutation in the *ER*α gene. Estrogen action on the skeleton is thought to occur mainly through the action of the nuclear receptors ERα and ERβ. Recently, in vitro studies have shown that the G protein–coupled receptor GPR30 is a functional estrogen receptor (ER). GPR30-deficient mouse models have been generated to study the in vivo function of this protein; however, its in vivo role in the male skeleton remains underexplored. We have characterized size, body composition, and bone mass in adult male *Gpr30* knockout (KO) mice and their wild-type (WT) littermates. *Gpr30* KO mice weighed more and had greater nasal-anal length (*p* < .001). Both lean mass and percent body fat were increased in the KO mice. Femur length was greater in *Gpr30* KO mice, as was whole-body, spine, and femoral areal bone mineral density (*p* < .01). *Gpr30* KO mice showed increased trabecular bone volume (*p* < .01) and cortical thickness (*p* < .001). Mineralized surface was increased in *Gpr30* KO mice (*p* < .05). Bromodeoxyuridine (BrdU) labeling showed greater proliferation in the growth plate of *Gpr30* KO mice (*p* < .05). Under osteogenic culture conditions, *Gpr30* KO femoral bone marrow cells produced fewer alkaline phosphatase–positive colonies in early differentiating osteoblast cultures but showed increased mineralized nodule deposition in mature osteoblast cultures. Serum insulin-like growth factor 1 (IGF-1) levels were not different. These data suggest that in male mice, GPR30 action contributes to regulation of bone mass, size, and microarchitecture by a mechanism that does not require changes in circulating IGF-1. © 2011 American Society for Bone and Mineral Research.

## Introduction

Bone is a dynamic compartmentalized organ composed of mineralized, stromal, and hematopoietic marrow compartments. Estrogen (E) signaling plays a critical role in many mechanisms required for proper bone growth and metabolism. Estrogens are synthesized from androgen precursors by the aromatase enzyme and historically have been thought to act mainly through the actions of estrogen receptor α (ERα) and ERβ. The importance of the action of estrogens on the human male skeleton has been demonstrated convincingly in patients with aromatase (estrogen synthase) deficiency(1–7) and loss of signaling through the ERα receptor.(8) Male patients deficient in either the aromatase enzyme or ERα have exhibited low bone mass and delayed growth plate closure. Mouse models of aromatase or ERα deficiency have shown that estrogens are also important in the regulation of bone mass and metabolism in male mice.(9–11)

Our understanding of the complexity of estrogen action has been expanded by the recent identification of the orphan G protein–coupled receptor GPR30 as an ER. In 2005, two groups showed that 17β-estradiol binds to and signals through it, leading to the designation of GPR30 as GPER1 in International Union of Pharmacology nomenclature.(12,13) GPR30 is a Gs-coupled seven-pass transmembrane protein originally cloned by multiple groups in the late 1990s.(14–18) In vitro it has been found to be activated by estrogen and G1, a GPR30-specific agonist,(19) and to induce cAMP elevation, intracellular calcium mobilization, and transactivation of epidermal growth factor receptors (EGFRs).(20–24) These signaling events may originate from GPR30 localized to the plasma membrane(21,22,24) or within an intracellular membrane compartment.(23,25) Initial human tissue expression analysis of GPR30 showed expression in skeletal muscle, heart, lung, liver, kidney, and brain,(14) with subsequent studies showing a similar distribution that included adipose in mice. Subsequently, expression has been shown in connective tissues such as cartilage and bone.(26,27) Expression in the human growth plate cartilage declines as puberty progresses.(26) In bone, GPR30 has been found to be expressed in osteoblasts, osteocytes, and osteoclasts.(27) In immortalized rat calvarial preosteoblasts, Runx2, a critical regulator of osteoblast cell differentiation, was shown to upregulate *Gpr30* gene expression. This resulted in increased cellular proliferation in these osteoblast progenitors.(28) Combined, the results of this study and the expression analysis strongly suggest a role for GPR30 in skeletal metabolism.

The in vivo function of GPR30 has been explored only recently in genetically defined mouse models. Four such models of GPR30 deficiency have been created using either homologous recombination to insertionally disrupt the coding exon(29,30) or Cre-lox technology to remove it.(31,32) Skeletal biology has been studied in a limited fashion. Martensson and colleagues reported a small age-dependent decrease in crown-rump and femur lengths, measures of skeletal growth, in *Gpr30* KO female mice but not males. In follow-on studies using these mice, Windahl and colleagues were able to show that estradiol treatment of ovariectomized mice reduced longitudinal skeletal growth, as measured by femur length, and decreased growth plate height in WT not *Gpr30* KO mice.(33) Male mice were not studied in this gonadectomized estradiol treatment paradigm. From these reports, it is clear that the in vivo role of GPR30 in the male skeleton remains underexplored. Therefore, we undertook studies to better understand the in vivo role of GPR30 in the male skeleton. We determined the effect of *Gpr30* knockout on growth plate proliferative activity, bone mass, and structure in male *Gpr30* null mice. Further, we studied in vitro differentiation of osteoblast and osteoclast precursors. All studies were performed using a previously developed mouse model in which the *Gpr30* gene locus is completely disrupted.(29)

## Materials and Methods

### Animals

The University of Texas Southwestern Medical Center Institutional Animal Care and Use Commitee (IACUC) approved all protocols. The animals were maintained in a controlled environment of 12 hours of light/12 hours of dark cycles at an ambient temperature of 22°C. Standard irradiated rodent chow and water were provided *ad libitum*. Mice carrying a *Gpr30* null mutation were created by targeted disruption of the gene with a Neo cassette and have been described previously.(29) In our laboratories, the mutation is maintained on a hybrid C57Bl6/J and 129SvEvTac background. Breeding nonsibling animals heterozygous for the null mutation generated 4-month-old homozygous null males and WT littermates.

### Morphometric measurements

Body weight and nasal-anal (NAL) length measurements were determined at the time of sacrifice. Mice were anesthetized via intraperitoneal injection of tribromoethanol (Avertin) (1.25% w/v, 0.2 mL/10 g of body weight) prior to measurement. NAL was measured using digital calipers (Mitutoyo Corporation, Kawasaki, Japan). Femur length and midshaft diameters were determined at the time of animal euthanization. Femoral midshaft diameters were measured at the inferior edge of the linea aspera.

### Dual-energy X-ray absorptiometry (DXA)

Whole-body densitometry (*n* = 18 WT, *n* = 22 *Gpr30* KO) was performed using the PIXIMUS densitometer (GE Lunar Corporation, Madison, WI, USA) equipped with software Version 2.10. By convention, the head was excluded from all regions of interest used in whole-body analyses. Total tissue mass was computed using a region of interest that excluded the head. Body fat (g) was calculated from the software computed percent body fat multiplied by the total tissue mass. Lean tissue mass (g) was calculated by subtracting bone mineral content (g) and fat mass (g) from the total tissue mass. Whole-body, lumbar spine, and femur areal bone mineral density (aBMD) measures were determined from the whole-body scans using the appropriate regions of interest.

### Growth plate labeling

BrdU labeling was used as an in vivo assay of growth plate proliferative activity (*n* = 8 WT, *n* = 9 *Gpr30* KO). BrdU (100 µL of a 70 µg/µL solution) was loaded into a 1003D Alzet osmotic pump (Cupertino, CA, USA) according to the manufacturer's instructions. Three days prior to euthanization of the mice, the pump was implanted intraperitoneally. An effective dose of 7 mg of BrdU per animal was administered.(34) Femurs for BrdU immunostaining were processed, embedded in paraffin, and sectioned in the University of Texas Southwestern Medical Center Molecular Pathology Core facility. Tissue sections were probed subsequently with an anti-BrdU Immunohistochemistry Kit (Oncogene, San Diego, CA, USA) according to the manufacturer's instructions. BrdU^+^ chondrocytes were determined microscopically at 200× magnification and scored as positive cells per 100 cells counted.

### Static and dynamic histomorphometry

To label the skeleton, tetracycline HCL (Sigma, St Louis, MO, USA) in aqueous solution (0.03 mg/g) was given i.p. at 10 and 4 days prior to euthanization. The left tibia was harvested for histomorphometry and stored in 70% ethanol until analysis (*n* = 9 WT, *n* = 8 *Gpr30* KO). After dehydration in a graded series of alcohol, the bones were processed undecalcified in methyl methacrylate as described previously.(35) Then 10-µm-thick sections were obtained in the longitudinal plane using a Reichert Polycut E microtome (Leica Microsystems, Bannock, IL, USA). Histomorphometric examination was performed via computer monitor on images captured from an Aus Jena microscope equipped with a video camera (Optronics, Goleta, CA, USA) and histomorphometry software (Bioquant Nova II, Nashville, TN, USA). Static measurements of osteoid indices and cellular parameters were performed on toluidine blue–stained sections. Measurements on trabecular bone were taken at a distance of at least 1 mm from the growth plate to prevent inclusion of primary spongiosa. Fluorochrome-based indices of bone formation were measured in unstained sections. Specimens were analyzed blinded to genotype. The terminology used to define the measured parameters is that recommended by the Histomorphometry Nomenclature Committee of the American Society for Bone and Mineral Research.(36) Abbreviations in the text are OS/BS (osteoid surface/bone surface), Ob.S/BS (osteoblast surface/bone surface), MS/BS (total mineralizing surfaces (single + double)/bone surface), BFR (bone-formation rate surface referent), Oc.S/BS (osteoclast surface/bone surface), and ES/BS (eroded surface/bone surface). BFR was calculated using ½ single labeled surfaces plus all double-labeled surfaces.

### Micro–computed tomographic (µCT) imaging

µCT imaging of the right femur (*n* = 9 per genotype) was performed using a Siemens Inveon CT/PET Multimodality system (Siemens Medical Solutions). Imaging was performed at 80 kV and 500 mA with a focal spot of 58 µm. Under high magnification, the effective pixel size was 11.34 µm. Reconstructed images were analyzed on an Inveon Research Workplace (Siemens Medical Solutions) using manufacturer-supplied software. Trabecular bone regions of interest (ROIs) were drawn approximately 180 µm from the growth plate and extended proximally 200 pixels along the cortical wall. The trabecular bone was segmented from the bone marrow and analyzed to determine the trabecular bone volume fraction (BV/TV), trabecular bone surface area per unit volume (BSA/BV), trabecular thickness (Tb.Th.), trabecular number (Tb.N.), and trabecular spacing (Tb.Sp.). Trabecular pattern factor (TPF), a measure of the connectedness of the trabecular structure within an ROI, also was calculated. The smaller the TPF index, the more connected is the trabecular bone.(37) Midshaft cortical wall thickness (Ct.Th.) was measured on a coronal projection image. Four measurements were taken perpendicular to the cortical wall on either side of the shaft and averaged.

### Serum IGF-1 levels

Blood was obtained by closed cardiac puncture at the time of euthanization. Serum was harvested from blood by centrifugation following clot formation and stored at −80°C to prevent protein degradation prior to analysis. Total IGF-1 serum levels (*n* = 15 per genotype) were measured after acid-ethanol extraction using an IGF-1 RIA (DSL-2900, Diagnostic Systems Laboratories, Webster, TX, USA) according to manufacturer's instructions.

### Cell culture

#### In vitro osteoblastogenesis

The proximal and distal ends of femurs were removed aseptically. Marrow cells were harvested by centrifugation through the bone at 10,000*g*. Harvested marrow cells were cultured in 12-well plates containing osteogenic medium [15% fetal bovine serum (Atlanta Biologicals, Norcross, GA, USA) in α-MEM (Invitrogen, Carlsbad, CA, USA) plus 5 mM β-glycerol phosphate (Sigma) plus 1 mM ascorbate 2-phosphate (Wako Chemicals, Richmond, VA, USA)]. For colony-forming units fibroblast (CFU-F) assays, cells were seeded at a density of 0.6 × 10^6^ cells per well and 1 × 10^6^ cells per well for colony-forming units osteoblast (CFU-Ob) assays. One-half the medium was replaced with fresh medium every 3 days. The cultures (CFU-F) were terminated at 7 days and stained for alkaline phosphatase expression (Sigma Kit 86R-1KT). Mineralized nodule formation (CFU-Ob) was allowed to proceed by culturing the cells to confluence (usually total of 14 to 20 days). Nodules then were stained with 40 mM alizarin red (S), pH 10.8, and dissolved in dH_2_O (MP Biomedicals, Solon, OH, USA). Quantification of CFU-F and CFU-Ob cultures was conducted by counting macroscopic colonies and alizarin red (S)^+^ nodules, respectively. The assays were repeated three times.

#### In vitro osteoclastogenesis

Bone marrow cells were plated in osteoclastogenic medium [10% FBS (Atlanta Biologicals) in α-MEM (Invitrogen) plus 5 ng/mL of macrophage colony-stimulating factor (M-CSF; R&D Systems, Minneapolis, MN, USA) plus 50 ng/mL of sRANKL (R&D Systems)]. The cells were seeded in a 24-well plate at a density of 2.5 × 10^5^ cells per well. One-half the medium was replaced with fresh medium every 3 days. Cultures were terminated on day 7 of culture and stained for tartate-resistant acid phosphatase (TRACP) according to the manufacturer's directions (Sigma Kit 387A-1KT). Osteoclasts were identified as multinucleated TRACP^+^ cells. The assays were scored by averaging the number of osteoclasts per low-power field in at least three fields per well. The assay was repeated three times.

### Statistical analysis

Data are expressed as mean ± SEM. Student's *t* test was used to determine statistical significance. In all instances, *p* < .05 was taken to be statistically significant.

## Results

### *Gpr30* inactivation causes increased body size and growth plate proliferation without changes in circulating IGF-1

**Fig. 1:**
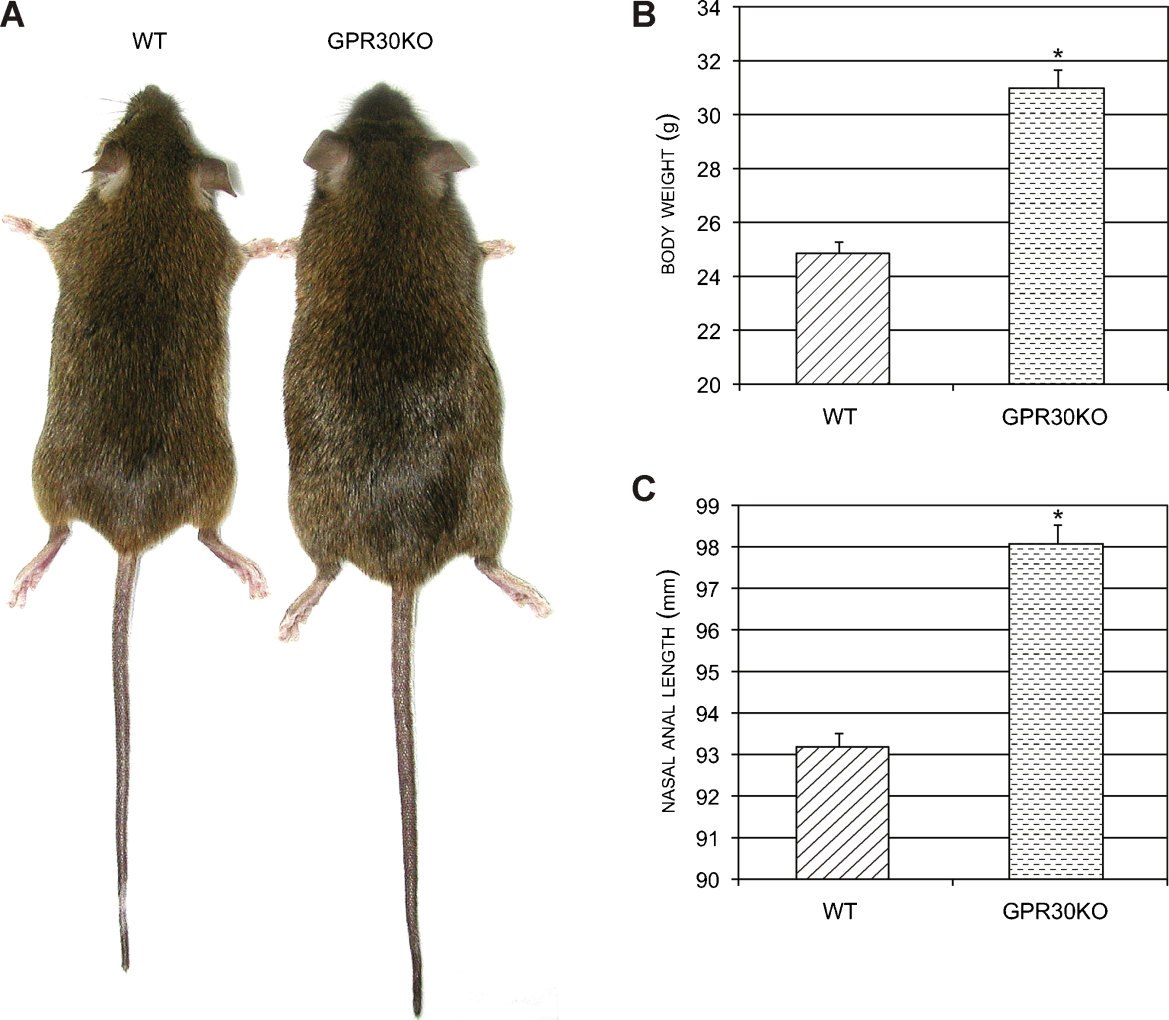
*Gpr30* KO mice have increased nasal-anal length and body weight at 4 months of age. (*A*) Representative image of 4-month-old WT and *Gpr30* KO mice. (*B*) Body weight of WT and *Gpr30* KO mice. (*C*) Nasal-anal lengths (NALs) of WT and *Gpr30* KO mice. *p < .0001 versus WT.

**Fig. 2:**
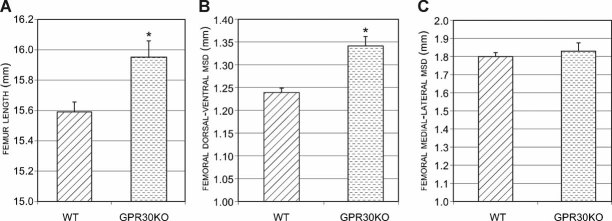
*Gpr30* KO mice have increased femoral size. (*A*) Femur length of WT and *Gpr30* KO mice. (*B*) Midshaft dorsal ventral femoral diameter (MSD) of WT and *Gpr30* KO mice. (*C*) Midshaft mediolateral femoral diameter of WT and *gpr30* KO mice. *p < .01 versus WT.

*Gpr30* KO mice had an increased body weight (30.98 versus 24.85 g, *p* < .0001; Fig. 1*A,B*) at 4 months of age. The NAL of the *Gpr30* KO mice was significantly higher than in WT males (98.07 versus 93.19 mm, *p* < .001; Fig. 1*C*). Appendicular skeletal growth was assessed by femoral measurements. Femoral length in *Gpr30* KO mice was increased when compared with WT mice (15.95 versus 15.59 mm, *p* < .01; Fig. 2*A*). Femoral midshaft diameter (MSD), a measure of femoral radial growth, showed an increase of 7% in dorsal-ventral growth in Gpr30 KO mice was observed when compared with WT littermates (1.34 versus 1.24 mm, *p* < .001; Fig. 2*B*). However, there was no statistical difference in mediolateral growth (1.83 versus 1.80 mm; Fig. 2*C*). Interestingly, serum IGF-1 levels were not different between the two genotypes (253 ± 15.31 versus 247.67 ± 31.08 ng/mL).

**Fig. 3:**
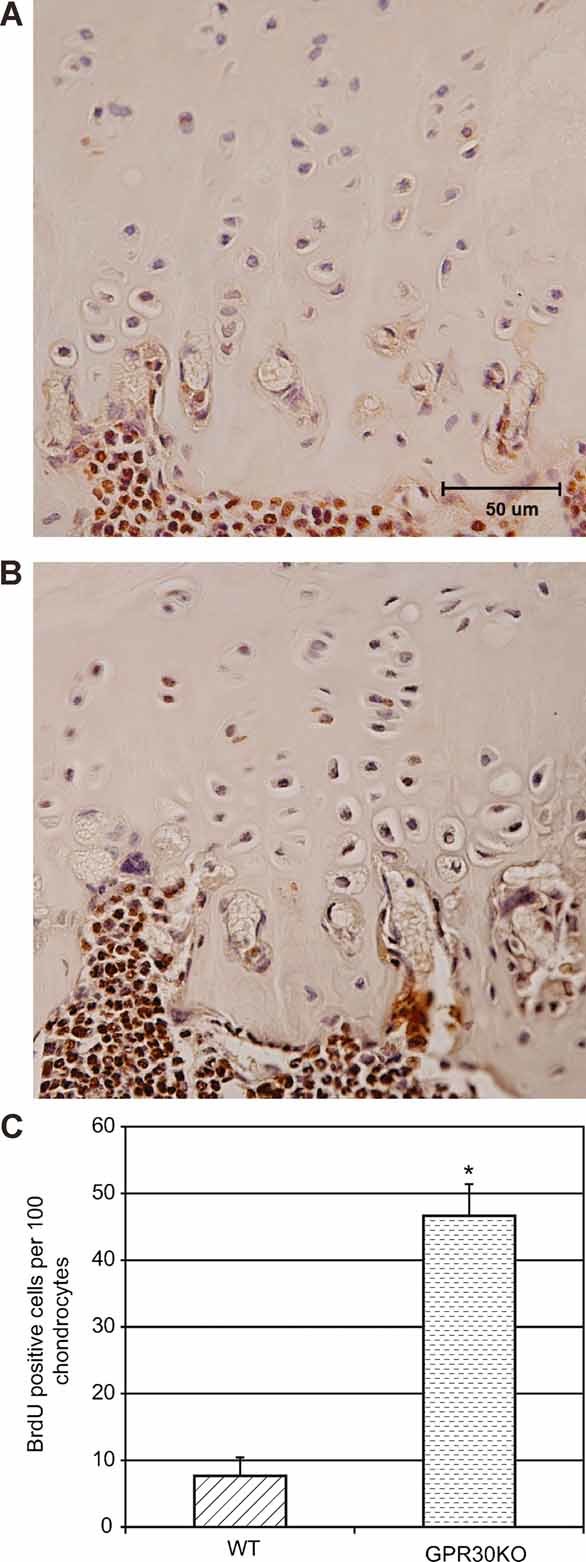
*Gpr30* KO mice have higher proliferative activity within the growth plate by anti-BrdU immunostaining. (*A*) Representative image of anti-BrdU staining within the WT femoral growth plate. (*B*) Representative image of anti-BrdU staining within the *Gpr30* KO femoral growth plate. (*C*) Microscopic count of BrdU^+^ chondrocytes. *p < .05 versus WT.

We hypothesized that the increased long bone growth seen in male *Gpr30* KO mice might be caused by increased growth plate proliferation. To test this hypothesis, immunohistochemistry was performed on distal femoral growth plates following in vivo labeling with BrdU. *Gpr30* KO mice had an increased number of BrdU^+^ chondrocytes per column (Fig. 3, *p* < .05). Staining differences were particularly apparent in the hypertrophic chondrocytes.

### *Gpr30* KO male mice have increased axial and appendicular bone mass by DXA

DXA was used to determine bone mineral density (BMD) of WT and *Gpr30* KO mice at 4 months of age. At this age, whole-body aBMD was elevated in *Gpr30* KO mice (53.88 versus 47.93 mg/cm^2^, *p* < .01; Table 1). In addition, these mice showed increased spinal (62.51 versus 54.19 mg/cm^2^, *p* < .001; Table 1) and femoral BMD (78.08 versus 68.38 mg/cm^2^, *p* < .001; Table 1). Lean body mass (23.41 versus 19.9 g) and percentage body fat (13% to 17.1%) were increased significantly in the *Gpr30* KO mice (Table 1).

**Table 1:** Bone Mass and Body Composition, MicroCT Analysis, and Histomorphometry

	WT	*Gpr30* KO
DEXA analysis		
BMD (mg/cm^2^)		
Whole body	47.93 ± 0.74	53.88 ± 0.69^a^
Spine	54.19 ± 1.19	62.51 ± 1.31^a^
Femur	68.38 ± 1.68	78.08 ± 1.47^a^
Body composition		
%Body fat	13.0 ± 0.45	17.1 ± 1.25^b^
Lean mass (g)	19.9 ± 0.39	23.41 ± 0.34^a^
MicroCT of trabecular bone and cortical thickness^f^		
BV/TV (%)	43 ± 2.1	58.5 ± 3.1^b^
BSA/BV (µm^−1^)	40 ± 3.9	29.16 ± 1.8^b^
Tb.Th. (µm)	55.1 ± 1.6	70.7 ± 4.3^b^
Tb.N. (µm^−1^)	7.8 ± 0.2	8.3 ± 0.1^c^
Tb.Sp. (µm^−1^)	74.19 ± 4.68	49.88 ± 3.52^d^
TPF (µm^−1^)	3.8 ± 0.8	-4.7 ± 1.2^d^
Ct.Th. (µm)	187.7 ± 1.7	227.8 ± 5.7^d^
Histomorphometry of cancellous bone^f^		
OS/BS	3.8 ± 0.8	9.1 ± 2.5^c^
Ob.S/BS	3.6 ± 0.8	8.7 ± 2.3^c^
MS/BS	15.6 ± 1.8	29.2 ± 4.5^e^
BFR	35 ± 6	64 ± 10^e^
Oc.S/BS	1.2 ± 0.2	1.9 ± 0.2
ES/BS	4.7 ± 0.6	4.7 ± 0.7

a *p* < .0001

b *p* < .01

c *p* = .06

d *p* < .001

e *p* < .05 vs. WT.

f Abbreviations are defined in “Materials and Methods.”

### GPR30-deficient male mice have thickened, more connected trabecular bone and thicker femoral cortical bone by µCT analysis

**Fig. 4:**
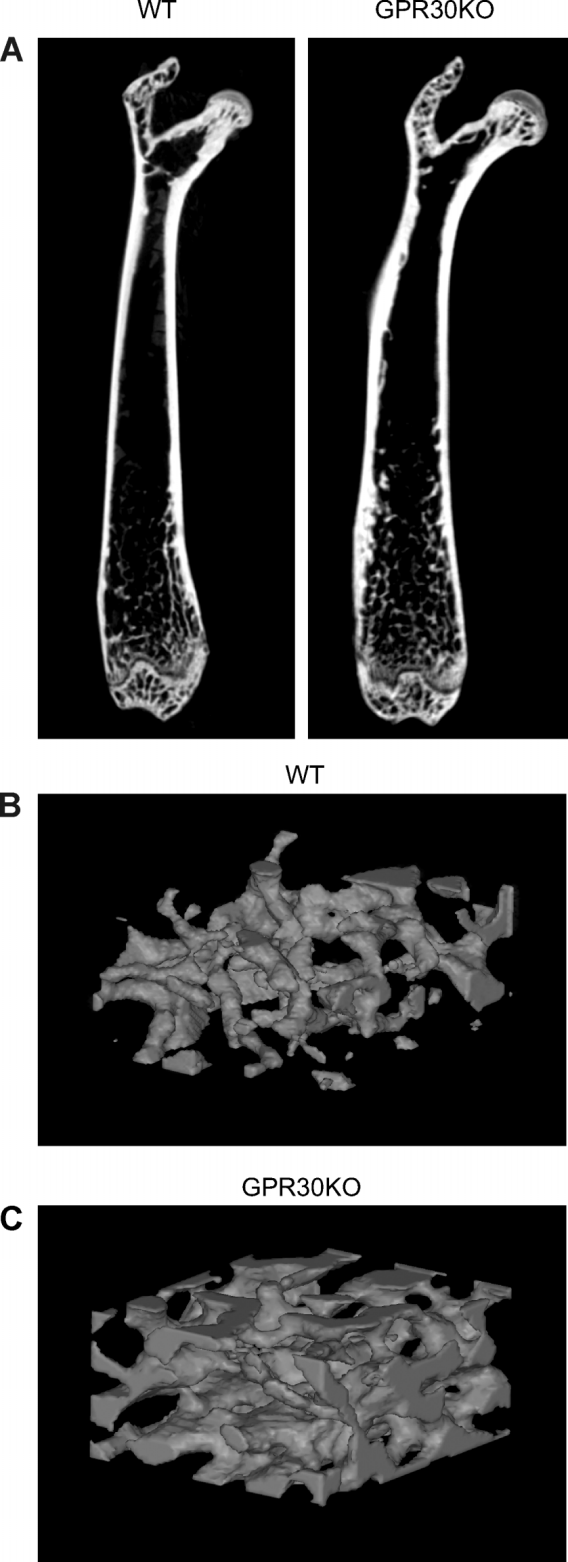
*Gpr30* KO mice have increased trabecular bone volume and connectivity in the distal femur. (*A*) Coronal section of WT and *Gpr30* KO femurs showing an increase in trabecular bone and cortical thickness. Images not to scale. (*B*) Representative 3D rendering of the distal femoral trabecular network of WT mice. (*C*) Representative 3D rendering of the distal femoral trabecular network of *Gpr30* KO mice.

µCT scanning of the distal femur was used to evaluate the effect of GPR30 deficiency on trabecular bone. Male *Gpr30* KO mice showed increased trabecular bone volume, connectivity, and trabecular thickness and decreased trabecular spacing in the distal femur (Table 1 and Fig. 4). BV/TV (%) was increased in the *Gpr30* KO mice (58.5% versus 43.0%, *p* < .01; Table 1). Trabecular thickness (70.72 versus 55.05 µm, *p* < .06; Table 1) was increased in the *Gpr30* KO mice, with an associated decrease in trabecular spacing (49.88 versus 74.19 µm^−1^, *p* < .001; Table 1). The more connected trabecular bone network of the *Gpr30* KO mice was reflected in the negative TPF, whereas the WT mice had a positive TPF (–4.7 versus 3.8, *p* < .001; Table 1).

### GPR30 deficiency causes increased mineralized surface, as measured by histomorphometry

**Fig. 5:**
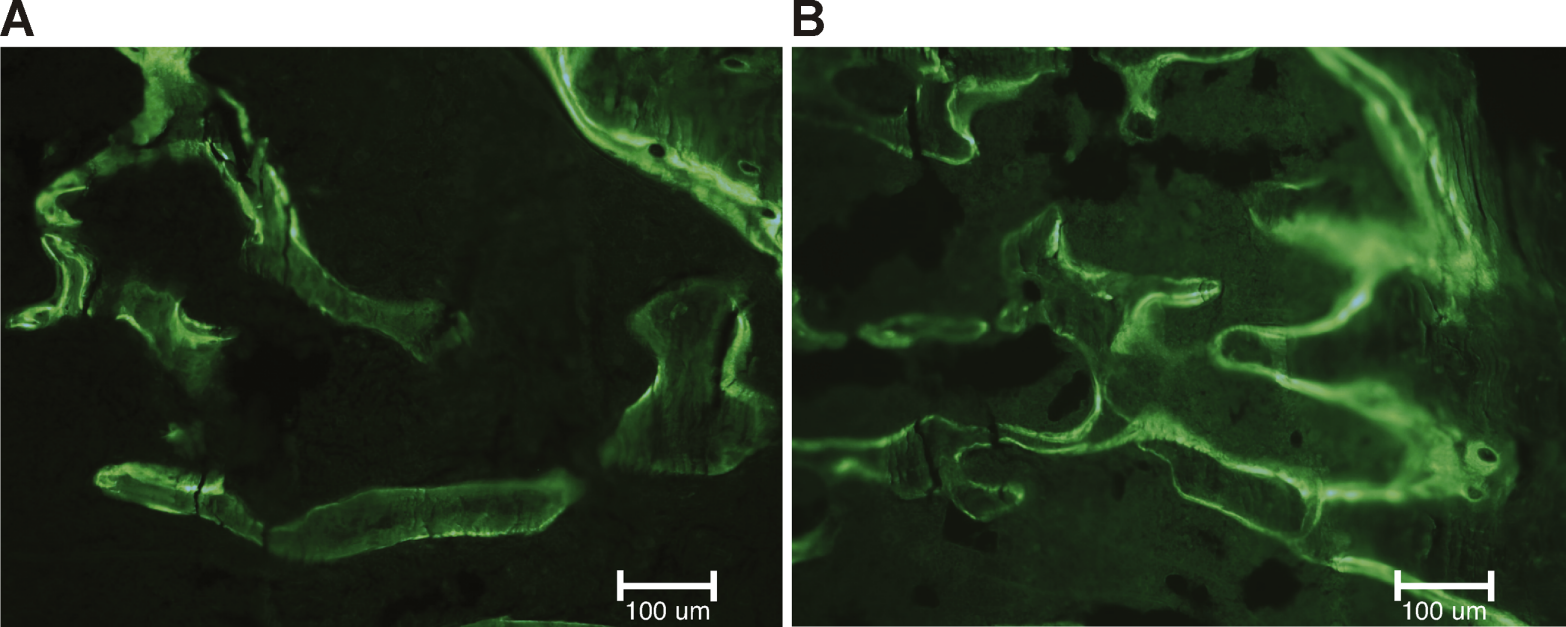
GPR30 deficiency results in increased mineralized surface in tibial cancellous bone. (*A*) Representative microphotograph of tetracycline-labeled cancellous bone in WT mice. Both single- and double-labeled surfaces are present. (*B*) Representative microphotograph of tetracycline-labeled cancellous bone in Gpr30 KO mice. There is a greater extent of labeled surface compared with WT mice. Magnification bar = 100 µm.

Static and dynamic histomorphometric analysis of the proximal tibia was used to determine the effect of *Gpr30* inactivation on cellular surfaces and mineralization in cancellous bone. Dynamic measurements revealed a significant increase in mineralized surface (MS/BS) in *Gpr30* KO mice (15.6% versus 29.2%, *p* = .02; Table 1 and Fig. 5) and BFR (35 versus 64 µm^3^/µm^2^/yr, *p* = .04). There was a strong trend toward increased osteoid (OS/BS) and osteoblastic (Ob.S/BS) surfaces in *Gpr30* KO mice (*p* = .06 versus WT mice; Table 1). There was no significant change in osteoclast (Oc.S/BS) or eroded (ES/BS) surfaces (Table 1).

### Gpr30 inactivation has stage-specific effects on in vitro osteoblast differentiation

**Fig. 6:**
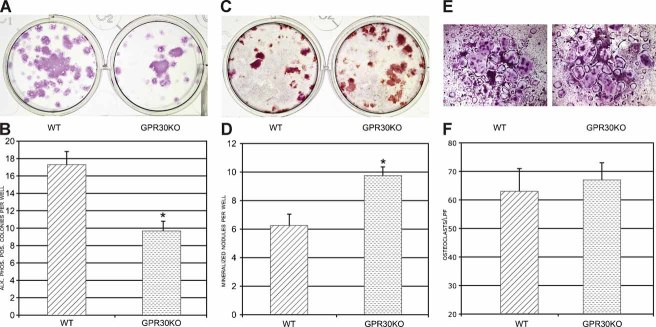
*Gpr30* KO osteoprogenitors show decreased colony number in early osteoblast differentiation but increased mineralized nodules in late-stage differentiation. In vitro osteoclastogenesis is unaffected by GPR30 deficiency. (*A*) Representative image of CFU-F colony staining for alkaline phosphatase from WT and *Gpr30* KO cultures. (*B*) Quantization of macroscopic CFU-F alkaline phosphatase^+^ colonies. (*C*) Representative image of mineralized nodules (CFU-Ob) stained with alizarin red (S) from WT and *Gpr30* KO cultures. LPF = low-power field. (*D*) Quantization of macroscopic mineralized nodules produced in CFU-Ob cultures. (*E*). Representative images of osteoclast formation induced by sRANKL and M-CSF stimulation of WT and Gpr30KO bone marrow cells. (*F*) Quantization of osteoclast formation. *p < .05 versus WT. The images are representative results from an experiment repeated three times.

To assess the effect of Gpr30 inactivation on cell autonomous osteoprogenitor differentiation, bone marrow–derived cells were cultured in vitro under osteogenic differentiation conditions. In the early culture period (day 7), there was a pronounced decrease in alkaline phosphatase positivity, consistent with a decrease in osteoblast precursor number or proliferation (Fig. 6*A, B*). On the other hand, late-stage cultures, usually 15 to 20 days, showed an increased number of mineralized nodules in the Gpr30 KO cultures (Fig. 6*C, D*).

## Discussion

GPR30 expression has been shown in human(26,27) and mouse bone (Ford and Oz, unpublished results), although its function in bone is only beginning to be explored. Gene deletion of the receptor provided a model to determine its role in male skeletal homeostasis. This report demonstrates for the first time in male mice that inactivation of GPR30 signaling causes abnormalities in body size, bone mass, and bone microarchitecture. *Gpr30* KO BMD was increased on DXA scans, and µCT evaluation of the distal femur revealed an increase in cortical thickness and in trabecular bone thickness, volume, and connectivity. This increased bone mass was associated with increased mineralized surface by quantitative dynamic histomorphometry. In vivo BrdU labeling revealed increased proliferative activity in the femoral growth plate of 4-month-old *Gpr30* null mice, suggesting that the receptor is important to cessation of growth plate proliferative activity.

Previous *Gpr30* transgenic and KO mouse models have been constructed to study the in vivo function of this protein.(29–32) Wang and colleagues created a *Gpr30* KO by insertional mutagenesis using homologous recombination to insert a Neo cassette into exon 3.(29) These investigators focused on thymic biology to show that GPR30 contributes to estrogen-induced thymic atrophy and thymocyte apoptosis but not developmental blockade of thymocytes, which seems to be mediated by ERα. Subsequent studies on these mice by Haas and colleagues showed that *Gpr30* deletion results in loss of the normal abrogation of vasoconstrictor-induced changes in vascular tone and increased visceral fat.(38) Isnee and colleagues reported on *Gpr30*-*lacZ* mice in which homologous recombination was used to replace exon 3 of the *Gpr30* locus by the *lacZ* reporter cassette.(30) In support of a role for GPR30 function in endothelial cells, the authors found lacZ expression in vascular beds of small arteries in multiple organs, including the heart, reproductive tract, and breast. They also found decreased levels of CD4^+^ and CD8^+^ T cells in the blood, which is consistent with increased the T-cell apoptosis reported by Wang and colleagues. In contrast to the mice generated by Wang and colleagues, the authors did not observe increased visceral fat. Otto and colleagues have reported *Gpr30* KO mice generated using Cre-lox technology.(31) The study placed emphasis on the reproductive tract, for which the authors reported no phenotypic abnormality in fertility, in agreement with phenotypes observed in other models of GPR30 deficiency. Studying the effects of estradiol therapy on the uterus and mammary gland, they observed no difference in the response of 10-week-old castrated WT and *Gpr30* KO mice. The lack of an observed effect could reflect no role for GPR30 in the responses studied, that the dosing regimen did not reflect normal physiologic fluctuation in serum E levels in animals, or that the function of traditional ERs is dominant at the concentrations used. Martensson and colleagues also reported a *Gpr30* KO mouse created by using Cre-lox technology to delete exon 3.(32) These investigations placed emphasis on metabolic and cardiovascular phenotypes. Abnormalities in glucose tolerance, blood pressure, and estradiol-stimulated insulin release were observed in female but not male knockouts. All combined, it is clear that previous studies using genetically altered *Gpr30* mice have emphasized immunologic, cardiovascular, metabolic, and reproductive phenotypes.

Before now, skeletal studies have been published for only one of the mouse models. Martensson and colleagues showed that genetic inactivation of GPR30 caused decreased crown-rump and femur lengths in 3-month-old female *Gpr30* KO mice but reported that there was no effect on the skeleton of male mice.(32) Subsequently, using the same mice Windahl and colleagues showed that, as in WT mice, ovariectomy-induced cortical and trabecular bone loss could be prevented in estrogen-treated female *Gpr30* KO mice, suggesting that GPR30 is not necessary for the prevention of bone loss after castration; however, unlike in WT mice, E treatment did not reduce growth plate height in the *Gpr30* KO mice.(33) Studies of male mice were not reported. These results indicate that estradiol action through GPR30 is compartment-dependent and required for normal estrogenic response in the growth plate of female mice. The sexually dimorphic impact of GPR30 deficiency is reminiscent of other models of deficient estrogen action.(9,11) As in our studies reported here, serum IGF-1 levels were not altered in their model. On the other hand, the lack of an observed male skeletal phenotype in their model of GPR30 deficiency contrasts with our observations of increased bone mass, body and femur length, and growth plate activity. The different results obtained with the models may be related to factors such as age, genetic background, diet (e.g., phytoestrogen content), or technology used to create the mice. In the mice studied by Martensson and colleagues and Windahl and colleagues, the mutation is carried on a mostly B6 background, whereas our mice were of a hybrid 129/B6 background. It is possible that genetic loci outside the *Gpr30* locus modify the phenotypes in a strain-dependent fashion. Another possibility relates to the impact that mutations of the *Gpr30* locus may have on an overlapping locus, *C7orf50* (GeneID 84310, Entrez Gene database). This open reading frame is transcribed in the opposite direction and codes for a hypothetical 194-amino-acid protein of unknown function. In both models, intronic sequence is altered, whether by deletion (Martensson model) or by insertional disruption (our model). Such alterations in introns may affect expression of proteins.(39) One might speculate that *C7orf50* expression is differently affected in the two models and that it has some role, direct or indirect, in bone homeostasis. Even if the two *Gpr30* KO mouse models differ with respect to impact on *C7orf50*, the sexually dimorphic effect of the knockout in the Martensson model still remains unexplained. Concern also has been raised about the presence of the Neo cassette in our model.(30) While we cannot rule out the theoretical possibility of nontarget effects of the Neo cassette, the fact that others have observed similar effects on early osteoblast proliferation and suppression of chondrogenesis (proliferation was not specifically measured) using completely different systems gives us confidence in the fidelity of our results.(28,40) We should point out it also has been reported that the Cre-lox approach used by the other authors may result in unrecognized chromosomal rearrangements.(41) Whatever is the explanation for the phenotypic differences between the two models, it is clear from results of both groups that GPR30 is a critical receptor in regulation of the growth plate.

It is now widely accepted that estrogens regulate longitudinal bone growth. In human males, it is believed the pubertal growth spurt is initiated by estrogens.(42) On the other hand, the lack of growth plate fusion in aromatase deficient (ArKO) and *ER*α KO patients established that estrogen action through ERα causes termination of long bone growth through growth plate fusion. Unlike in humans, growth plate fusion does not occur in mice, but growth plate closure, characterized by a marked decrease in proliferative activity, does. Despite this species difference, the fundamentals of chondrocyte proliferation should be consistent across species. The increased BrdU labeling observed in the GPR30-deficient growth plate showed a clear increase in proliferation of chondrocytes and is consistent with the observed increased long bone growth phenotype in *Gpr30* KO mice. It establishes a role for GPR30 in chondrocytes, in which it serves as a “brake” to slow chondrocyte proliferation. Furthermore, in male rodent knockouts of estrogen action, only the *Gpr30* KO recapitulates the persistent growth plate activity that leads to the continued linear growth observed in humans.(43) With this observation, it is reasonable to infer that, at least in male mice, GPR30 action functions to limit longitudinal skeletal growth. Whether it plays the same role in humans awaits case reports of patients with GPR30 deficiency; however, it is noteworthy that GPR30 expression in the human has been shown to change with age during puberty, and it is postulated to regulate longitudinal bone growth.(26) Conceivably, at low doses, when E is postulated to stimulate long bone growth,(42) the conventional ERs are dominant. On the other hand, at higher doses of E, the action of GPR30 may come into play,(44) thereby limiting or terminating long bone growth.

In contrast to our results, previous studies on *Ar* KO(9,10) or *ER*α KO(45) male mice have shown decreased femoral length or no change in the complete *ER*α KO mice.(11) This is opposite of the finding in most *Ar* KO patients and in the one reported *ER*α KO patient. The molecular basis for this species difference is unclear; however, *Ar* KO and *ER*α KO mice have high testosterone levels, which are known to have antiproliferative effects on chondrocyte proliferation.(46) *Gpr30* KO mice do not have elevated testosterone as a confounding factor.(32) Therefore, the model will be a valuable resource in unraveling estrogen's action on the growth plate.

To begin to unravel the cellular mechanisms that account for the high BMD phenotype, we undertook differentiation studies of bone marrow cells cultured under osteogenic and osteoclastogenic conditions. Despite previous studies reporting GPR30 expression in osteoclasts, we did not observe any changes in osteoclastic surface, eroded surface, or in vitro differentiation from precursors cells. Therefore, its function remains to be determined in osteoclasts. On the other hand, our in vitro results demonstrated a bifunctional role for GPR30 during osteoblast differentiation. Early-stage osteoblastic in vitro differentiation cultures showed a decrease in alkaline phosphatase^+^ colony number, consistent with decreased proliferation. The results are consistent with a previous report on mouse osteoblast progenitors that showed *Gpr30* is a Runx2-responsive gene and acts in a promitogenic fashion through a Cdk pathway.(28) Taken together, these observations are consistent with a model in which GPR30 signaling stimulates proliferation of immature osteoblasts. Moreover, considering our results showing increased proliferation in the GPR30-deficient growth plate, it seems that GPR30 functions in bone as a pro- or antiproliferative factor in a cell-specific fashion. This aspect of GPR30 activity has been discussed recently in cancer cell lines.(47) In contrast to results from these cell autonomous assays, in vivo we not observe a change in osteoblast surface/number. It should be noted that we did not directly measure osteoblast proliferation or apoptosis in vivo and that within the complex bone microenvironment, other factors may compensate for the decreased proliferation in cultured GPR30-deficient osteoblasts. Late-stage cultures, under osteogenic conditions, showed increased mineralized nodule formation by cells deficient in GPR30. This suggests that GPR30 somehow functions to limit matrix mineralization. Loss of this aspect of GPR30 function may be a mechanism through which the in vivo increased BMD phenotype was achieved. Indeed, dynamic histomorphometry studies showed higher indices of mineralization (MS/BS) and osteoid formation (OS/BS) in *Gpr30* KO mice. Increased skeletal loading from the greater body weight conceivably could contribute to high bone mass in *Gpr30* KO mice. While the relationship between body mass and bone mass is complex,(48) we would expect that increased mechanical loading of the tibias from greater weight to affect osteoclasts, leading to, if anything, decreased resorption relative to formation in a load-bearing limb.(49) On histologic evaluation (Table 1), we did not observe a decrease in osteoclast or eroded surface. Therefore, we do not think that body mass is the driver of the bone phenotype. While the underlying molecular mechanisms remain to be defined, the increased bone mass points to a potential therapeutic opportunity, inhibition of GPR30 to increase BMD.

A number of the signaling pathways downstream of *Gpr30* have been defined mainly in cells other than bone, particularly breast cancer cells. This has been the subject of excellent recent reviews, but suffice it to say that these include rapid activation of MAP kinase, SRC-like tyrosine kinase, and adenylate cyclase.(44,50,51) The latter is of particular interest in bone biology because cAMP is the second messenger used by the most well-known GPCRs in bone, namely, the parathyroid hormone/parathyroid hormone–related protein (PTH/PTHrP) receptor and the prostaglandin receptors EP1 to EP4. The PTH receptor acts in part by stimulating cAMP production, and receptors EP2 and EP4 act by stimulating cAMP production. Furthermore, like GPR30, all are expressed in osteoblasts, and chondrocytes. Therefore, one might postulate the existence of functional redundancy between GPR30 and these other GPCRs in bone. Thus it might be surprising that *Gpr30* KO mice have a bone phenotype. Several considerations are important here. First, the duration and intensity of the signal generated by the receptors may differ. Even the effect of PTH itself on bone differs according to dosing regimen. PTH, when given in a pulsatile fashion, is anabolic to bone. On the other hand, continuous high levels of PTH, as in hyperparathyroidism, cause bone loss. Second, the outputs and targets/consequences of the signaling pathways may differ. For instance, PTH has been shown to inhibit osteoblast proliferation, whereas estrogen stimulates it. Of note and consistent with our current results are recent in vitro studies reported by Teplyuk and colleagues that showed that GPR30's action in early osteoblastogenesis is proproliferative,(28) suggesting that the proliferative effects of estrogen may be mediated in part by GPR30. Finally, since it is known that there are different cyclases, even if GPR30 shares second messengers with other GPCRs in bone, the receptors' signaling pathways may involve different cyclases.

In summary, this study demonstrates that GPR30 is important to regulation of proliferation within the male mouse growth plate and to bone volume within cancellous bone. The underlying mechanisms apparently do not require changes in circulating IGF-1. This is the first male mouse model with deficient estrogen action that reproduces the long bone growth abnormality seen in human *Ar* KO or *ER*α KO patients. Understanding the molecular mechanisms by which GPR30 regulates osteoblast function and proliferative activity within the growth plate not only will increase our understanding of basic bone biology but also may lead to novel therapies for short stature and low bone mass.
